# Roles of small RNAs in the effects of nutrition on apoptosis and spermatogenesis in the adult testis

**DOI:** 10.1038/srep10372

**Published:** 2015-05-21

**Authors:** Yongjuan Guan, Guanxiang Liang, Penelope A. R. Hawken, Irek A. Malecki, Greg Cozens, Philip E. Vercoe, Graeme B. Martin, Le Luo Guan

**Affiliations:** 1 UWA Institute of Agriculture and School of Animal Biology, University of Western Australia, 35 Stirling Highway, Crawley, WA 6009; 2 Department of Agricultural, Food and Nutritional Science, University of Alberta, Edmonton, Alberta, T6G 2P5, Canada; 3 Nuffield Department of Obstetrics & Gynaecology, University of Oxford, Oxford OX3 9DU, UK; 4 Department of Animal Sciences, University of Stellenbosch, Matieland 7600, South Africa; 5 School of Anatomy, Physiology and Human Biology, University of Western Australia, 35 Stirling Highway, Crawley, WA 6009

## Abstract

We tested whether reductions in spermatozoal quality induced by under-nutrition are associated with increased germ cell apoptosis and disrupted spermatogenesis, and whether these effects are mediated by small RNAs. Groups of 8 male sheep were fed for a 10% increase or 10% decrease in body mass over 65 days. Underfeeding increased the number of apoptotic germ cells (P < 0.05) and increased the expression of apoptosis-related genes (P < 0.05) in testicular tissue. We identified 44 miRNAs and 35 putative piRNAs that were differentially expressed in well-fed and underfed males (FDR < 0.05). Some were related to reproductive system development, apoptosis (miRNAs), and sperm production and quality (piRNAs). Novel-miR-144 (miR-98), was found to target three apoptotic genes (*TP53*, *CASP3*, *FASL*). The proportion of miRNAs as a total of small RNAs was greater in well-fed males than in underfed males (P < 0.05) and was correlated (r = 0.8, P < 0.05) with the proportion of piRNAs in well-fed and underfed males. In conclusion, the reductions in spermatozoal quality induced by under-nutrition are caused, at least partly, by disruptions to Sertoli cell function and increased germ cell apoptosis, mediated by changes in the expression of miRNAs and piRNAs.

Spermatogenesis is regulated by a specific and complex genetic program that is susceptible to many disorders that can induce infertility^1^. In addition to disease situations, spermatogenesis can be affected by environmental factors, such as nutrition, that operate through normal physiological processes to profoundly change testicular mass and the efficiency of sperm production, thus affecting sperm output^2^,^3^. In the male sheep, for example, 2 months of mild under-nutrition leads to a reversible reduction in testis mass, impaired spermatogenesis, a reduction in the numbers of sperm produced per unit mass of testis (efficiency of spermatogenesis), and a reduction in sperm motility^4^. These effects seem to be mediated, at least partly, by changes in the activity of the Sertoli cells^5^. However, a thorough understanding of the effects of nutrition on spermatogenesis is elusive because of the complexity of the process of spermatogenesis, and the breadth of the spectrum of endocrine and paracrine signals that coordinate events from the initiation of meiosis through the differentiation of germ cells to the generation of mature spermatozoa^6^.

Disruption of spermatogenesis by under-nutrition also seems to involve the pathways of apoptosis^7^, a crucial event in many physiological and pathological conditions^8^ that was detected long ago in the seminiferous epithelium and is thought to be an important determinant of sperm output^9^. The efficiency of spermatogenesis depends on the total number of cells at successive stages of spermatogenesis^10^, so is probably regulated by programmed cell death. Indeed, factors that disrupt spermatogenesis can induce apoptosis in the testis – for example, suppression of FSH activity, which reduces Sertoli cell proliferation and germ cell number, leads to loss of germ cells through apoptosis rather than through a decrease in proliferation^11^,^12^. Similarly, selenium deficiency results in apoptosis of germ cells by arresting the cell cycles^13^. On the other hand, a relationship between the rate of apoptosis and the level of nutrition has not been reported, not even for the sheep, a model in which acute but reversible responses to nutrition are well documented^14^. The present study was therefore designed to test whether nutrition affects germ cell apoptosis in the testis of the sexually mature male sheep. It was also designed to investigate the molecular regulatory processes involved in the responses to changes in nutrition.

Among the regulators of spermatogenesis^15^,^16^, small RNAs, including microRNAs (miRNAs) and PIWI-interacting RNAs (piRNAs), have recently become prominent. miRNAs are small (~22 nucleotides) endogenous RNAs that negatively regulate gene expression by targeting the 3’-untranslated region (3’UTR)^17^ and/or coding region^18^ of mRNAs. It has been reported that a global loss of miRNAs, in germ cells or Sertoli cells, is detrimental for male fertility^19^. By contrast, piRNAs are longer (26–32 nt) than miRNAs and can bind to PIWI, a spermatogenesis-specific protein belonging to the Argonaute protein family^20^,^21^. The main function of piRNAs is to guide PIWI protein to repress the transposable elements that protect genomic integrity^22^. In addition, piRNAs derived from mRNAs play a role in the regulation of gene expression^23^. To date, piRNAs have been mainly found in the testis, suggesting their role specific to spermatogenesis^24^. Indeed, we have a confluence of hypotheses here because it has been reported that some miRNAs are crucial for the process of apoptosis^25^. For example, miR-98 expression is reduced during apoptosis^26^, miR-14 is a dose-dependent suppressor of apoptosis, miR-278 antagonizes apoptosis^27^, and transient inhibition of miR-21 in germ cell cultures increases the rate of apoptosis^19^.

As important regulators of spermatogenesis and germ cell apoptosis, miRNAs and piRNAs should help explain the effects of nutrition on sperm production and sperm quality in males, for example, male sheep. In the present study, we used sexually mature male sheep as a model because the reversible effects nutrition on testis mass, sperm production and sperm quality are well documented^28^, thus providing a solid foundation for studying the roles of small RNAs. We therefore 1) profiled miRNAs and piRNAs in sheep testis; 2) investigated the relationships among miRNA functions, spermatogenesis and germ cell apoptosis, particularly during responses to nutrition; and 3) explored the potential for gene-derived piRNAs as regulators of spermatogenesis in the testis of the sexually mature sheep.

## Materials and methods

The experimental protocol was approved by the Animal Ethics Committee of the CSIRO Centre for Environment and Life Sciences, Floreat, Western Australia (Project No.1202). All the methods followed the approved protocol and were conducted in accordance with the approved guidelines.

### Animals and treatments

From late autumn to mid-winter, 16 Merino male sheep were housed in individual pens in a building with windows allowing good penetration of natural light (CSIRO Floreat, Western Australia, latitude 31o59’S). On entry, the rams were 24 months old, weighed 65.7 ± 4.7 kg, and had a scrotal circumference of 31.8 ± 2.5 cm. During a 3-week acclimatization period, they were all fed daily with 750 g oaten chaff (8.4% crude protein; 8.0 MJ/Kg Metabolisable Energy) and 200 g lupin grain (35.8% crude protein; 13.0 MJ/Kg Metabolisable Energy). They were then allocated into two dietary treatments (‘high’ and ‘low’) with the groups balanced for training success to semen collection, body mass, scrotal circumference, temperament, poll-horn type, and sperm quality (the percentage of live and motile sperm, sperm concentration). The high diet was designed to allow the animals to gain 10% body mass over 65 days whereas the low diet was designed to allow 10% loss in body mass. At the start of the treatment period, individual daily allowance was adjusted to provide two dietary groups: animals fed the High diet were offered 1.2 kg oaten chaff plus 0.3 kg lupin grain; animals fed the Low diet were offered 0.51 kg chaff and 0.13 kg lupin grain. Every week, the animals were weighed and the amount of feed offered to each individual was adjusted to ensure achievement of target change in body mass. The outcomes for body and testis growth, and for sperm production, have been reported before^4^.

### Tissue collection and preservation

After 65 days of treatment, the animals were killed with an intravenous overdose of sodium pentobarbitone, and the testes were immediately removed, dissected and weighed. Three samples were chosen from the top, middle and bottom of both testes; those from the right testis were snap-frozen in liquid nitrogen and stored at –80 ˚C for total RNA preparation; those from the left testis were washed with 0.9% saline and then fixed with 4% paraformaldehyde for 6 h, dehydrated and processed for routine embedding in paraffin wax for histological analysis.

### Evaluation of apoptosis

Terminal deoxynucleotidyl transferase mediated dUTP nick-end labeling (TUNEL) was performed under the instruction of the ApopTag plus peroxidase *in situ* Apoptosis Detection Kit (Chemicon International, USA). Briefly, deparaffinized tissue sections (top part of left testes) were incubated with proteinase K (20 μg/ml), subjected to 3% H_2_O_2_ at 37 ˚C for 30 min to inhibit endogenous peroxidase, and then incubated with equilibration buffer at room temperature for 1 min. Each section was incubated with TdT (terminal deoxynucleotidyl transferase) at 37 ˚C for 1 h and then washed in stop/wash buffer for 10 min. The sections were incubated in anti-Digoxigenin Peroxidase Conjugate at room temperature for 30 min and were stained with diaminobenzidine (DAB) as a peroxidase substrate. After counterstaining with methyl green, numbers of TUNEL-positive cells per tubule were counted in 50 tubules per animal with the aid of a light microscope. All counting procedures were performed ‘blindly’.

### Isolation of RNA and Reverse transcription

The trizol method was used to isolate total RNA^29^ with the quality and quantity of RNA determined by Agilent 2100 Bioanalyzer (Agilent Technologies, Santa Clara, CA) and Qubit 2.0 Fluorometer (Invitrogen, Carlsbad, CA), RNA with an integrity number (RIN) higher than 7.0 was used for further analysis. A high capacity RNA-to-cDNA kit from Applied Biosystems was used for reverse transcription.

### Quantitative real-time PCR (qRT-PCR)

QRT-PCR was performed using SYBR Green (Fast SYBR® Green Master Mix; Applied Biosystems) to detect mRNA relative expression, with primers specifically targeting sheep fas ligand *(FASL)*, tumor protein p53 (*TP53)* and caspase3 (*CASP3*) genes designed using NCBI primer blast (http://www.ncbi.nlm.nih.gov/tools/primerblast/index.cgi?LINK_LOC=BlastHome). Primer sequences for *GAPDH* were obtained from a published source (Table S1)^30^. Fluorescence signal was detected with StepOnePlus™ Real-Time PCR System (Applied Biosystems). The primer specificity was confirmed by PCR amplification, agarose gel electrophoresis and amplicon sequencing. The total volume of each reaction contained 10 μl Fast SYBR Green Master Mix (Applied Biosystems), 1 μl of each primer (20 pmol/μl), 7 μl of nuclease-free water, and 1 μl of DNA template (50 ng/μl). Samples were measured in triplicate using the following program: 95 °C for 10 min for initial denaturation and then 40 cycles of 95 °C for 20 s, followed by annealing/extension for 30 s at 60 °C. Analysis of melting curves was used to monitor PCR product purity. Previous work had shown that the level of expression of *GAPDH* is relatively high and consistent during testicular development in the sheep, indicating its suitability as a housekeeping gene expressed at similar levels in somatic and germ cell populations^30^. The ΔΔCt method was used to analyze relative gene expression^31^. The same sample was always used as calibrator. The gene expression (ΔCt value) was calculated on the basis of quantification cycles (Ct) (ΔCt = Ct _target gene_ – Ct _GAPDH_). The levels of expression of apoptosis-related genes were calculated in relation to the calibrator (ΔΔCt = mean ΔCt _sample_ – mean ΔCt _calibrator_). Relative expression (RQ) was calculated using StepOnePlus™ Real-Time PCR System (Applied Biosystems) and the formula: RQ_target gene_ = 2^–ΔΔCt^.

### Small RNA library sequencing

In each sample, 1.0 μg of total RNA was used to construct miRNA libraries with a unique index using the TruSeq Small RNA Sample Preparation kit (Illumina, San Diego, CA) according to the manufacturer’s instruction. PCR amplification was performed for 11 cycles and gel purification was used to individually purify libraries with unique indices. Quantitative real-time PCR (qRT-PCR) was performed for library quantification using the StepOnePlus™ Real-Time PCR System (Applied Biosystems, Carlsbad, CA) and KAPA SYBR Fast ABI Prism qPCR kit (Kapa Biosystems, Woburn, MA). Individual libraries were then pooled for sequencing at Génome Québec (Montréal, Canada) using the HiSeq 2000 system (Illumina) to generate 50 b single reads. All the reads were demultiplexed according to their index sequences using CASAVA version 1.8 (Illumina) and reads that did not pass the Illumina chastity filter were removed from the dataset. The small RNAs sequencing reads with good quality were subjected to 3’ adaptor sequence trimming, and all reads were mapped by blastn to the non-coding RNA sequences (Rfam) to remove sequences belonging to tRNA, snoRNA, rRNA, and other non-coding RNAs.

### Identification of miRNAs

The miRNAs were identified using the methods outlined in a previous study^32^. Briefly, known miRNAs were identified by mapping the filtered 18 to 25 nt sequences to miRBase (release version 20), a searchable database of published miRNA sequences and annotation^33^. All reads from 16 libraries were pooled to predict novel miRNA using miRDeep2 based on the reference genome sequence of OAR3.1 (http://www.livestockgenomics.csiro.au/). Small RNA sequences with a miRDeep2 score higher than 5 and read numbers larger than 10 were defined as novel miRNAs in sheep. The novel miRNA precursor sequences were then combined with the known miRNA precursor sequences to form a new custom reference database. Sequencing reads from different samples were mapped to the new custom reference database to get the read number for the known and novel miRNAs for each sample. Homologous miRNAs were identified by the method described previously^34^.

The conservation of known miRNAs was analyzed based on the definitions for “highly conserved”, “conserved”, and “poorly conserved” from Targetscan^35^. More specifically, highly conserved miRNAs are those conserved across most vertebrates; conserved miRNAs are those conserved across most mammals, but usually not beyond placental mammals; poorly conserved miRNAs are those not present in the above two groups. In this study, ovine-specific miRNAs were defined by using two criteria: 1) they belong to a poorly conserved group; 2) their seed region sequences have only been reported previously in sheep.

The genomic location of the miRNAs was searched for using the UCSC Genome Browser (http://genome.ucsc.edu/) based on the reference genome sequence of OAR3.1 (http://www.livestockgenomics.csiro.au/). The miRNA genes are distributed across chromosomes either individually, or in clusters. A cluster is a group of miRNA genes located within a short distance (10 Kb) on the same chromosome, based on the definition in the miRBase database (http://www.mirbase.org). In the present study, all the known and novel miRNAs were grouped into various clusters based on their genomic location.

### piRNA characterization

To identify piRNAs, sequencing reads that ranged from 26 to 32 nt were mapped to the ovine genome by Bowtie (version 1.0.1). Reads that could not be perfectly mapped to the genome were discarded, and the remainders were de-duplicated to unique sequences. The filtered unique reads were subjected to an online predictor (http://59.79.168.90/piRNA/analysis.php), that relies on the training sets from non-piRNA and piRNA sequences of five model species sequenced: rat, mouse, human, fruit fly and nematode, to predict piRNA candidates^36^. The positions in the ovine genome of these candidates were obtained by Bowtie and, to avoid confusion caused by multiple locations, only those with a single location were further analyzed. The piRNAs in each library were quantified by blastn and customized perl scripts. All the sequencing data were deposited in a publicly available Gene Expression Omnibus database (http://www.ncbi.nlm.nih.gov/geo/), the data are accessible through GEO accession number GSE62797.

### Identification of differentially expressed (DE) miRNAs and piRNAs

Differential expression (DE) of miRNA/piRNAs between the nutritional treatments was investigated using the bioinformatics tool, edgeR^37^, that utilizes a negative binomial distribution to model sequencing data. The expression of miRNAs/piRNAs in each library was normalized to counts per million reads (CPM) by the following formula: CPM = (number of miRNAs/piRNAs reads/total reads number per library) × 1,000,000. miRNAs/piRNAs with CPM > 5 in at least 50% of the samples were subjected to analysis of differential expression. Fold change (FC) was defined as the ratio (low diet/high diet) of the arithmetic means of CPM values. Significant differential expression was accepted when false discovery rate (FDR) was < 0.05 based on Benjamini and Hochberg multiple-testing correction^38^, as well as FC < 0.67 or > 1.5^39^.

### Validation of miRNA expression using stem-loop qRT-PCR

The TAQMAN miRNA assay was used to validate miRNA expression following the manufacturer’s recommendation (Applied Biosystems). In brief, cDNAs were reverse transcribed from 10 ng total RNA, using 5 X specific miRNA RT primer, and then amplified using a 20 X TAQMAN miRNA assay. StepOnePlus™ Real-Time PCR System (Applied Biosystems) was used to detect the fluorescence signal. miRNAs with cycle threshold (Ct) values > 35 were considered as having not been expressed. In this study, U6 snRNA was used as an internal control^40^ and three biological replicates were performed. The 2^-ΔΔCt^ method was used to analyze the expression level and all statistical analyses were carried out using SPSS software (Version 20). One-way ANOVA was used to compare the groups, and P < 0.05 was considered significant. Data are expressed as Mean ± SEM.

### miRNA target prediction and functional analysis

TargetScan Release 6.0 (http://www.targetscan.org/)^41^ and miRanda (http://www.microrna.org/microrna/home.do)^42^ were used to predict the target genes for selected miRNAs. The 3’UTR sequences of genes from sheep were obtained from Ensembl Gene 75 *Ovis aries* genes (Oar_v3.1) (http://uswest.ensembl.org/). The predicted target genes by both TargetScan (default parameters)^43^ and miRanda (Total score ≥145, Total energy ≤ –10)^44^ for each miRNA were further analyzed through ingenuity pathway analysis (IPA; Ingenuity Systems, www.ingenuity.com). The significance of the predicted function in IPAs was determined using a corrected P value calculated by the Benjamini-Hochberg method (FDR)^38^. Threshold FDR < 0.05 and molecule number > 2 were used to enrich significant biological functions of each miRNA.

### miRNA target validation using dual luciferase reporter assay

The entire 3’UTRs of *TP53*, *BCL2-like 1*(*BCL2L1)*, *CASP3* and *FASL* were amplified from sheep genomic DNA by the method of PCR. All the primers are shown in Table S2. Both PCR products were cloned into the pmirGLO Dual-Luciferase miRNA Target Expression Vector (Promega) using the *Xho*1 and *Sal*1 restriction sites.

A sheep fetal testis cell line (ATCC® CRL-6546) was cultured in ATCC-formulated Dulbecco’s Modified Eagle’s Medium (Catalog No. 30-2002), supplemented with 10% fetal bovine serum (Gibco, Invitro-gen, Carlsbad, CA, USA), in a 37 °C incubator with 5% CO_2_. The 60 nM miR-98 mimics/miRNA mimic negative control (Ambion) was co-transfected with 200 ng luciferase reporter containing *BCL2L1*, *CASP3* or *FASL* 3’UTR using Lipofectamine 2000 reagent (Invitrogen) in 24-well plates. After transfection for 48 h, the Dual-Glo luciferase assay system (Promega) and SpectraMax M3 system were used to obtain readouts of firefly and Renilla luciferase. All the firefly luciferase readouts were normalized to their matching renilla luciferase readouts.

## Results

### Relationship between nutrition and apoptosis

**Figure 1:**
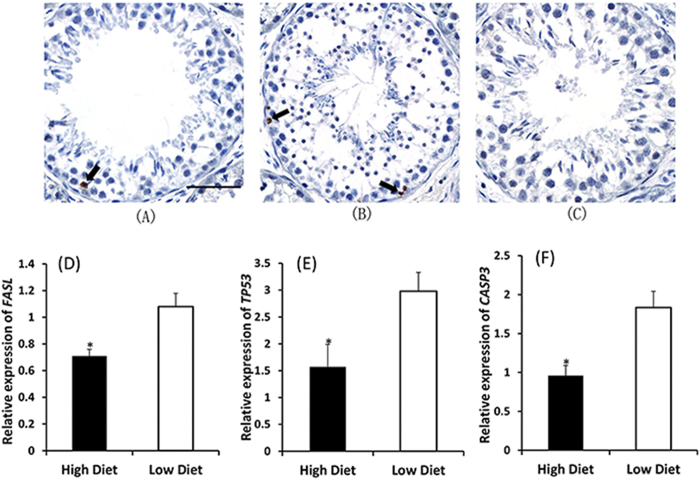
TUNEL-positive cells (arrows) detected in the testis in sexually mature sheep fed the High diet (**A**) or the Low diet (**B**) Negative control without terminal deoxynucleotidyl transferase (**C**) The scale bar represents 50 μm. (**D, E, F**): relative mRNA expression for apoptosis-related genes, normalized to *GAPDH*. Values are mean ± SE, N = 8 for each treatment. Significant effect of diet: *P < 0.05.

TUNEL-positive germ cells were observed in all treatments (Fig. 1A,B), but most were seen in the early stages of spermatogenesis (spermatogonia and spermatocytes) and none were seen amongst spermatids or spermatozoa. The number of TUNEL-positive germ cells per tubule was greater in underfed (1.4 ± 0.3) than in well-fed males (0.49 ± 0.06; P < 0.05). A relationship between under-nutrition and apoptosis was further supported by the expression of the apoptosis-related genes, *FASL*, *TP53* and *CASP3*, with all three showing greater expression in underfed sheep than well-fed sheep (P < 0.05, Figs. 1D,E,F).

### Profiling of small RNAs in the ovine testis

A total of 64 million high-quality small RNA reads were obtained from 16 testes (8 from each dietary group), with an average of 4.2 million reads per library (range: 1.2 million to 7.4 million). There was a bimodal length distribution with two peaks at 22 and 30 nt (Fig. S1). For the miRNAs class (18 to 25 nt), a total of 13 million reads were obtained, of which 10.4 million were mapped to the ovine genome. Mapping to Rfam database allowed us to remove 1.7 million reads that could be mapped to snoRNAs, snRNAs, tRNAs, rRNAs or other non-coding RNAs. Among the remaining 8.7 million reads, we identified 1.9 million unknown small RNAs, 5.5 million known and 1.3 million novel small RNAs, resulting in the identification of 110 known miRNAs and 194 putative novel miRNAs candidates from the ovine testis. All novel miRNA candidates were mapped to all vertebrate miRNAs in miRBase, a database of published miRNA sequences and annotation, to identify the homologues of novel miRNAs (Table S3). Based on the definition in Targetscan, among the 110 known and 194 novel miRNA candidates identified in sheep testis, 41 known and 62 novel miRNA candidates were highly conserved, 28 known and 21 novel miRNA candidates were conserved, and 36 known and 42 novel miRNA candidates were poorly conserved. From the poorly conserved group, 5 known and 69 novel miRNA candidates were sheep-specific and found in all of our animals. In the genomic context, 16 clusters of miRNAs were identified on 9 chromosomes and two large clusters comprising 40 miRNA precursors were identified on Chromosome 18 (Table S4).

piRNA sequences were predicted based on previously published databases, as described above. There were around 44 million reads of length 26 to 32 nt, of which 23.8 million reads mapped perfectly to the ovine genome. In total, 6 million reads representing 13567 putative piRNAs were identified and named with the prefix “oar-piR” followed by a number (data deposited in Gene Expression Omnibus). There were 13241 putative piRNA candidates mapping to unique loci, and these putative piRNAs were selected for direct comparison between the well-fed and underfed sheep.

### Identification of differentially expressed (DE) miRNAs and piRNAs

**Figure 2:**
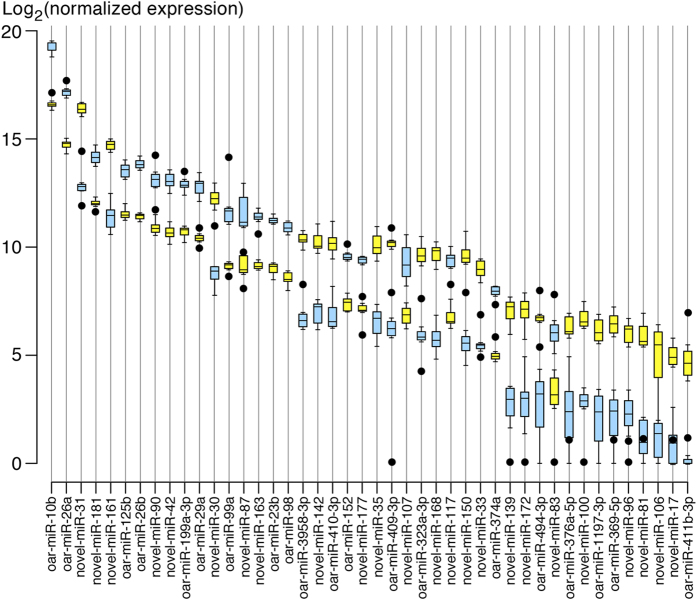
Box plot showing the differentially expressed (DE) miRNAs in testis from sexually mature sheep fed a high diet (yellow bar) and the low diet (blue bar). Central lines inside the boxes indicate median values, box width indicates 25% and 75% quartile ranges around the median, “T” indicates the maximum and minimum values, and black dots represent outliers. N = 8 for each treatment.

There were 44 DE miRNAs in testicular tissue from underfed and well-fed males, of which 21 were known and the rest were novel. Among all the DE miRNAs, 20 miRNAs including novel-miR-144 showed greater expression in underfed than in well-fed males (Fig. 2). In addition, the expression of DE miRNAs detected by the RNA-sequencing data reflected qRT-PCR expression results. Six known and 6 novel miRNAs were selected from the DE miRNAs and, for all of them, the qRT-PCR expression results were consistent with the sequencing data. For example, both the sequencing data and the qRT-PCR results showed that oar-miR-411b-3p was expressed at a lower level in underfed males than in well-fed males (Fig. S2). In addition, the expression of novel-miR-144 was down regulated in well-fed male sheep (Fig. S2).

For putative piRNAs, a total of 35 were DE in underfed and well-fed male sheep (Table S5), and among them, two (oar-piR-12568, oar-piR-6442) were derived from the 3’UTR of mRNAs (*FLVCR2, KRTAP10-2*). One putative piRNA, piR-9006, was derived from the 5’UTR of mRNA (*ATP2B4*), and 11 putative piRNAs were derived from introns within 7 genes (*MORF4L1*, *SNX5*, *STOX1*, *ENSOARG00000013508*, *FAH*, *CLEC16A*, *DCAF6*) (Table S6).

### Relationship between nutrition and top ten expressed miRNAs

The top ten expressed miRNAs were the same in tissues from underfed males and well-fed males, and their total expression did not differ between treatments (68% versus and 75% of the total miRNAs) (Fig. S3). However, expression of three of the top ten miRNAs did differ between treatments – specifically, oar-miR-10b and oar-miR-26a showed greater expression in underfed than well-fed males, whereas novel-miR-31 showed the opposite effect (Fig. 2).

### Relationship between nutrition and genomic location of miRNAs

There were two large clusters of miRNAs on Chromosome 18 and they included 10 miRNAs for which expression was significantly lower (FDR < 0.05, FC > 1.5) in underfed than well-fed males (Table S4).

### Relationships between miRNAs and piRNAs

**Figure 3:**
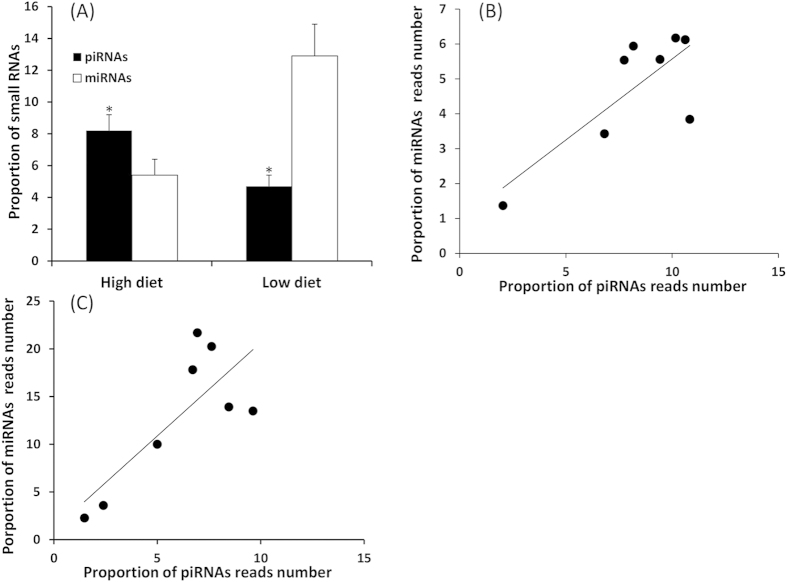
Proportions of putative piRNAs (black columns) and miRNAs (white columns) in each dietary treatment (**A**) Correlation between proportions of piRNAs and miRNAs in testis from sheep fed the High diet (**B**) or the Low diet (**C**) N = 8 for each treatment. The proportions of miRNAs or piRNAs were calculated as number of miRNAs or piRNAs reads divided by the total number of small RNA reads.

The proportion of miRNAs/piRNAs was defined by the ratio: miRNAs or putative piRNAs reads number /total small RNAs reads number. Interestingly, the proportion of putative piRNAs was greater than the proportion of miRNAs in well-fed males (P < 0.05, Fig. 3A) whereas the proportion of miRNAs was greater in underfed males (P < 0.05, Fig. 3A). In addition, there was a positive correlation between the proportion of miRNAs and proportion of putative piRNAs in testicular tissue from well-fed males (r = 0.8, P < 0.05, Fig. 3B) and underfed males (r = 0.8, P < 0.05, Fig. 3C).

### Functional analysis of miRNAs

The targets of the miRNAs were predicted with TargetScan and miRanda and it was found that 74 sheep-specific miRNAs would target 7783 genes. The function of these targeted genes was analyzed by IPA. The most common functions are listed in Table 1 where it can be seen that, in general, they are related to synthesis of lipids and hormones, to testis morphology, and production of sperm. Also, 3567 genes were predicted to be targeted by the miRNAs clustered on Chromosome 18. Their most common functions were related to testis morphology (P < 0.05).

**Figure 4:**
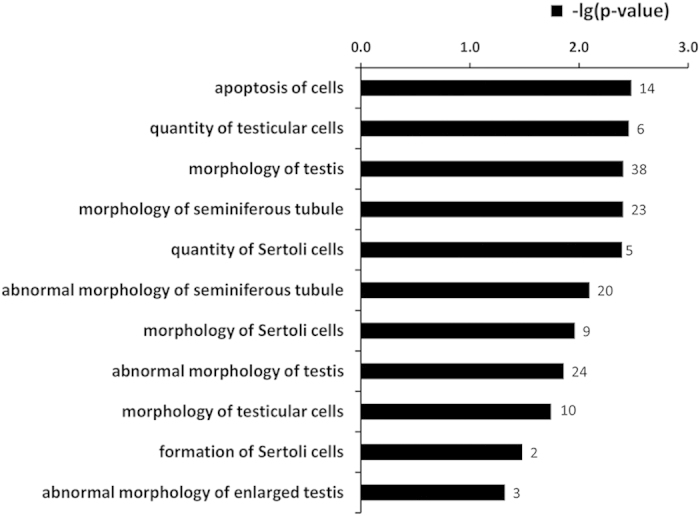
The top 11 functions of DE miRNAs related to development and function of the reproductive system. X-axis represents the –lg (p-value) and indicates the relevance of the function to the DE miRNAs, with a lower p-value (a higher value of – lg(p-value)) suggesting greater relevance.

In addition, 44 DE miRNAs were predicted to target 1,597 genes (data not shown), and a total of 14 functional categories were identified, including those involved in the development and function of the hematological and reproductive systems (Table 2). We identified 11 biological processes that were targeted within the category of development and function of the reproductive system, including apoptosis and Sertoli cell number (Fig. 4). Furthermore, IPA analysis revealed that these DE miRNAs were also involved in 76 signaling pathways, of which apoptosis signaling, germ cell-Sertoli cell junction signaling, Sertoli cell-Sertoli cell junction signaling are among the most relevant pathways (Fig. S4). To test our hypothesis that apoptosis explains the poor sperm quality in underfed animals, we further analyzed the DE miRNAs in the apoptosis-signaling pathway. We found that 12 genes involved in apoptosis could be targeted by 9 DE miRNAs, with novel-miR-144 targeting four of the apoptosis-related genes (*FASL*, *CASP3*, *BCL2L1*, *TP53*).

### Validation of predicted miRNA targets

**Figure 5:**
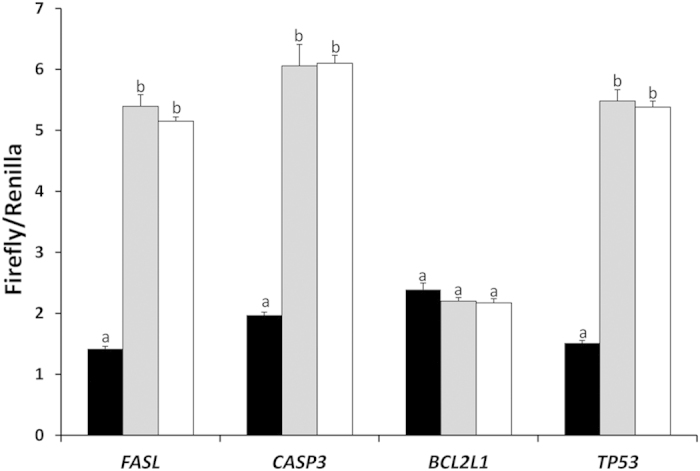
Normalized luciferase activity. Black column: co-transfection of novel-miRNA-144 mimics and reconstructed pmirGLO vector (containing 3’UTR of target genes). Grey column: co-transfection of novel-miRNA-144 mimics and pmirGLO vector (without 3’UTR of target genes). White column: co-transfection of miRNA mimics negative control and reconstructed pmirGLO vector (containing 3’UTR of target genes). Values are mean ± SE (N = 8 per treatment). a, b, c: different letters denote statistically significant differences within each target gene.

We validated *FASL*, *CASP3*, *BCL2L1* and *TP53* as direct targets for novel-miR-144 in a biological process using a dual luciferase reporter assay on a sheep fetal testis cell line. Co-transfection of novel-miRNA-144 mimics and pmirGLO vector containing 3’UTR of *TP53* decreased normalized luciferase activity by 72% compared to the pmirGLO vector no-insert control (P < 0.01, Fig. 5). There was no difference between the pmirGLO vector no-insert control and miRNA mimics negative control. Similar results were observed with *CASP3* and *FASL* for which there were 67% and 74% decreases compared with the no-insert control (P < 0.01, Fig. 5). However, normalized luciferase activity of pmirGLO that contained *BCL2L1* was not affected by novel-miR-144 (Fig. 5).

## Discussion

This is the first comprehensive description of small RNAs in sheep testis and, by combining these observations with bio-informatics analysis and experimental validation, in the context of an experimental model of reversible testis growth in the sexually mature males, we have been able to identify miRNAs and piRNAs that are associated with the control of testis function. Importantly, we have also shown how the expression of these small RNAs changes in response to under-nutrition in association with apoptosis in germ cells. Our findings strongly support the hypothesis that the decline in sperm production and sperm quality induced by under-nutrition^4^ in the sexually mature sheep are mediated at least partly by increased apoptosis in germ cells.

### Profile of small RNAs in sheep testis

Around 63% of the known miRNAs and 43% of the novel miRNAs that we detected are conserved or highly conserved, suggesting that their biological functions are conserved across species^45^. The present study also revealed 5 known and 69 novel miRNAs that are sheep-specific, most with functions focused on synthesis of lipid or hormones, or production of sperm. This differed from the conserved miRNAs, suggesting that sheep-specific miRNAs may represent an important source of novel functionalities during evolution, an idea previously raised for the human and the mouse^46^. The ten most highly ranked miRNAs are relatively well conserved across species – for example, four miRNAs (miR-143, let-7a, let-7f, miR-148a) in pig testis^47^ and two miRNAs (let-7a, let-7f) in human testis^48^ ranked within the ten most highly expressed miRNAs found in this study. This suggests that they play similar roles in the control of testis function for a variety of mammalian species. To our knowledge, the present study is the first to profile piRNAs in sheep testis. About 10% of the small RNAs were predicted to be putative piRNAs, similar to pigs (13%), mice (10%) and humans (9%)^20^,^48^,^49^. However, in contrast to miRNAs, the sequences of the putative piRNAs are weakly conserved among species^50^.

### Impact of under-nutrition on miRNAs and apoptosis

The predicted targets of the 44 miRNAs that were differentially expressed (DE) between well-fed and under-fed sheep were related to tissue morphology and development of the reproductive system. Among all the DE miRNAs, there were 24, including novel-miR-31, that were more highly expressed in well-fed sheep than in under-fed sheep. Novel-miR-31 is homologous with miR-34 which has been shown to enhance germ cell phenotype during the late stages of spermatogenesis in other species^51^. Under-nutrition was associated with greater expression of 20 miRNAs, including miR-99a, which reduces the expression of the tight-junction-related protein, ZO-1^5^,^52^.

The predicted targets of top ten expressed miRNAs were functionally related to diseases of the reproductive system, connective tissue function and development, tissue morphology and cellular growth and proliferation, suggesting a critical role in regulating reproductive performance. Under-nutrition did not affect the composition of the top ten, but did affect the expression of three of them - specifically, underfed males showed higher expression of oar-miR-10b and oar-miR-26a, and lower expression of novel-miR-31 (miR-34c), than well-fed males. The genes that were up-regulated in underfed males have been implicated in the induction of apoptosis (miR-26a)^53^ and testis dysfunction (miR-10b)^54^, whereas novel-miR-31(miR-34c), which was up-regulated in well-fed males, enhances germinal phenotypes in late spermatogenesis^51^. These roles are consistent with the conclusion that, during the loss of testis mass with under-nutrition, apoptosis is induced and spermatogenesis is disrupted.

We had hypothesized that increased apoptosis is one of the main causes of the negative effect of under-nutrition on sperm production^4^ and spermatogenic efficiency^5^. Underfed sheep had more TUNEL-positive germ cells and higher expression of apoptosis-related genes (*FASL*, *CASP3*, *TP53*)^55^,^56^,^57^ than well-fed sheep, thus supporting our hypothesis. Interestingly, TUNEL-positive germ cells were observed only in the early stages of spermatogenesis (spermatogonia and spermatocytes) and not in spermatids or spermatozoa. This observation agrees with reports for other species^58^,^59^,^60^, suggesting that spermatogenic cells are mostly eliminated before the first meiotic division. However, there are a few reports of apoptosis in spermatids or spermatozoa^61^. It is therefore possible that our TUNEL assay was not able to adequately label spermatids or spermatozoa due to either the compact nature of their DNA or their limited production of mRNA, generally thought to be necessary for apoptotic death^61^.

We focused our investigation on novel-miR-144 (homolog: miR-98) for two reasons: first, novel-miR-144 was up-regulated in underfed sheep; second, novel-miR-144 targets four of the apoptosis-related genes (*FASL*, *CASP3*, *TP53*, *BCL2L1*)^26^,^62^. Our working hypothesis, that the negative effects of under-nutrition on spermatogenic efficiency are mediated by increased apoptosis, is supported by studies in mice^63^ where the homologue to novel-miR-144 has been shown to be pro-apoptotic. However, our finding contradicts some previous reports showing that miR-98 is up-regulated in small-cell lung cancer^64^ and breast cancer^65^, two conditions associated with cell proliferation rather than apoptosis. We also found a positive correlation between expression of novel-miR-144 and the apoptosis-related genes, *FASL*, *CASP3* and *TP53,* and therefore contradict the conventional wisdom that the expression of miRNAs is negatively correlated with their target genes^66^. However, this type of positive correlation has been reported previously in mice^67^ and could be interpreted as miRNAs playing a role in homeostatic mechanisms that maintain stability within the organism. The underfed sheep had increased TUNEL staining indicating increased apoptosis, most due to increased expression of the apoptosis-related genes, *FASL*, *CASP3* and *TP53.* miRNAs are thought to ‘fine tune’ the physiological balance within an organism, so it is possible that the increased expression of novel-miR-144 was a response to the increase in apoptosis, rather than a cause of the apoptosis. Novel-miR-144 relationships are obviously complex, but we propose that miR-98, and thus its sheep homologue (novel-miR-144), is both pro-apoptotic and anti-apoptotic depending on the physiological condition of organism.

### Impact of nutrition on putative piRNAs

Of the 35 differentially expressed piRNAs found in well-fed and underfed sheep, 60% were derived from intergenic and repeated regions of the genome. Based on observations in pigs, these DE piRNAs may have specific germline functions, including the repression of transposons and other repetitive elements^20^. The remaining 40% of the piRNAs that were differentially expressed in the current study were derived from genes. It is difficult to predict the specific functions of the piRNAs identified in the present study because our current understanding of their function is limited and their sequence identities are poorly conserved among species. We therefore focused only on the gene-derived DE piRNAs and predicted their functions based on previous references – for example, feline leukemia virus subgroup C receptor-related protein 2 (FLVCR2), which produces piR-12568 and functions as a calcium transporter and affects reproduction and respiration^68^. In addition, P-type Ca^2+^-ATPase isoform 4 of the plasma membrane (ATP_2_B_4_), which produces piR-9006, is responsible for sperm motility^69^. Furthermore, five piRNAs (piR-10216, piR-10217, piR-10729, piR-10730, piR-10731) that showed higher expression in underfed than well-fed sheep, were derived from MORF4L1, a gene that is highly expressed during male meiosis and spermatogenesis^70^ and seems likely to play a crucial role in male reproduction. To date, the mechanism through which piRNAs regulate sperm production and sperm quality is not clear. However, there has been a suggestion that a piRNA pathway was active in Sertoli cells^71^ and, as we have shown, Sertoli cell function is reduced in underfed males^5^. We therefore expect that piRNAs affect male reproduction by regulating the function of Sertoli cells, a hypothesis that needs to be tested in further studies.

### Relationships between miRNAs and piRNAs

In well-fed males, the proportion of putative piRNAs, as a percentage of total small RNAs, was greater than the proportion of miRNAs. This relationship was reversed in underfed males. These observations suggest that nutrition has distinct, differential effects on the expression of small RNAs in sheep testis. This relationship could be explained by the higher rate of sperm production in well-fed males compared to underfed males^72^, because piRNAs are specifically expressed in germ line cells^73^. Interestingly, there was a positive correlation between the proportions of miRNAs and putative piRNAs in testicular tissue, for both underfed and well-fed males, indicating a synergistic relationship between these classes of small RNAs. Further studies are needed to test this hypothesis.

**Figure 6:**
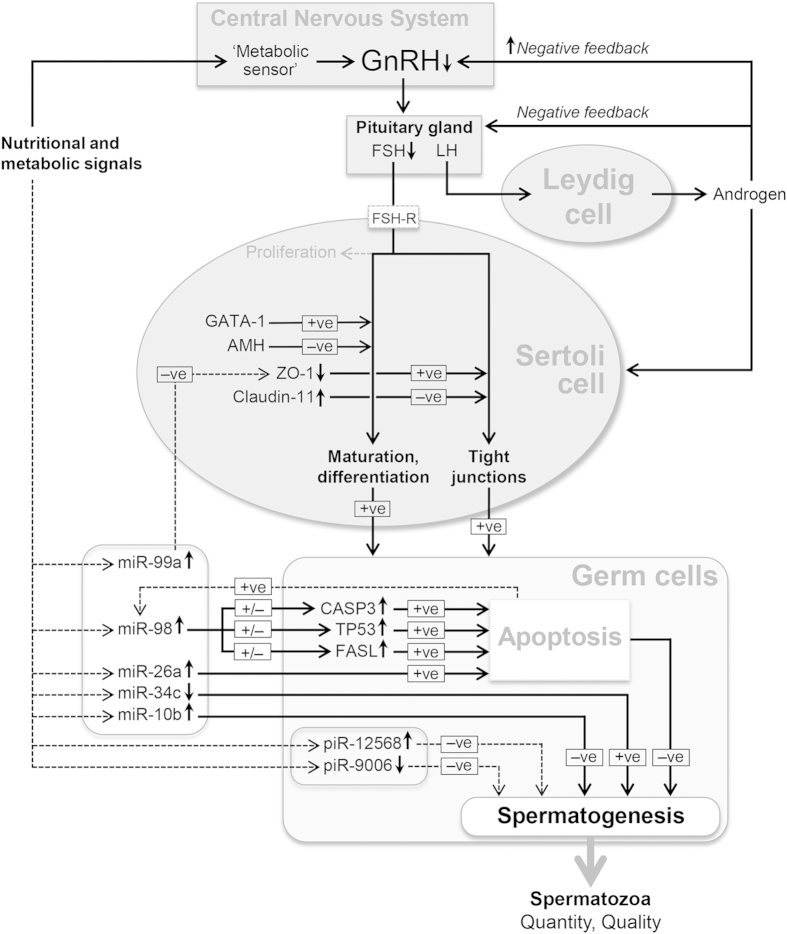
A working hypothesis of the mechanisms through which nutrition affects testis function in the sexually mature sheep, indicating roles that could be played by small RNAs. Stimulation is indicated by “+ve” and inhibition is indicated by “–ve”. The effects of under-nutrition on each regulatory factor are indicated by vertical arrows. Solid lines denote pathways supported by strong evidence whereas broken lines indicate pathways where the evidence is still accumulating. Nutritional and metabolic signals do not seem to affect the proliferation of Sertoli cells but they do affect Sertoli cell function, notably the organization of tight junctions and cellular maturation. The Sertoli cell responses, combined with nutrition-induced changes in germ cell apoptosis, affect the quantity and quality of spermatozoa produced. Several small RNAs are strongly affected by under-nutrition: miR-34c, miR-10b, piR-9006 and piR-12568 are directly associated with spermatogenesis; miR-98 and miR-26a regulate apoptosis of germ cells; miR-99a is predicted to regulate tight junctions by targeting ZO-1 expression. In addition, there are correlations between the proportions of miRNAs and piRNAs, although it is not clear whether this reflects mutual regulation, and there appears to be a feedback loop linking apoptosis and miR-98 expression.

In conclusion, under-nutrition is associated with increased germ cell apoptosis, as evidenced by increases in TUNEL-positive germ cells and in the expression of genes and miRNAs that are related to apoptosis. Furthermore, in underfed males, the differential expression of miRNAs and piRNAs is likely to help explain the negative effects of nutrition on spermatogenesis, spermatogenic efficiency and sperm quality. These conclusions, combined with the outcomes of research on the effects of nutrition on the reproductive axis of the male sheep^5^, led us to develop a working hypothesis that explains how nutrition affects testis function (Fig. 6). Specifically, under-nutrition leads to a reduction in FSH secretion^74^, a key hormone associated with the function of Sertoli cells and, significant changes in Sertoli cell function, including the disorganization of tight junctions and reversal of cell maturity^5^. The outcome is impaired spermatogenesis and reductions in the number of germ cells per unit mass of testis^5^, and increases in germ cell apoptosis that dramatically reduced the quantity and quality of spermatozoa^72^. Small RNAs seem to be involved in these processes. Specifically, miR-98 appears to regulate germ cell apoptosis by changing the expression of *CASP3, TP53* and *FASL* in parallel with the direct effects of miR-26. Similarly, changes in expression of miR-99a seem to affect the organization of Sertoli-cell tight junctions by targeting ZO-1^52^. The other small RNAs that we have detected (miR-26, miR-34c, miR-10b, piR-9006, piR-12568) are involved in the control of reproductive function in other species, so might also contribute to the effects of nutrition on spermatogenesis in the sexually mature sheep. The role of piRNAs in this process is far from clear, but the distinct differences between well-fed and underfed males in piRNA expression, and in the proportion of piRNAs as a total of small RNAs, suggest that they also play an important, possibly synergistic, role.

## Author Contributions

Y.G. and G.L. performed the molecular analysis, data analysis and draft writing; P.H., I.M. and G.B.M. contributed to animal study and data analysis; G.C. oversaw the TUNEL analysis; P.E.V. and L.L.G. contributed to interpretation of the molecular data. All authors contributed to manuscript writing.

## Additional Information

**How to cite this article**: Guan, Y. *et al.* Roles of small RNAs in the effects of nutrition on apoptosis and spermatogenesis in the adult testis. *Sci. Rep.* **5**, 10372; doi: 10.1038/srep10372 (2015).

## Supplementary Material

Supporting Information

## Figures and Tables

**Table 1:** Functions or diseases linked to 74 sheep-specific miRNAs detected in the testis of sexually mature sheep.

**Category**	**Function or Disease**	**p-Value**
Small Molecule Biochemistry	Synthesis of lipid	3.04E-03
Endocrine System Development and Function	Synthesis of hormone	8.24E-03
Lipid Metabolism	Synthesis of steroid	8.24E-03
Organ Morphology	Abnormal morphology of enlarged testis	1.12E-02
Reproductive System Development and Function	Abnormal morphology of enlarged testis	1.12E-02
Cell Morphology	Size of connective tissue cells	1.26E-02
Small Molecule Biochemistry	Steroidogenesis	1.33E-02
Endocrine System Development and Function	Steroidogenesis	1.33E-02
Reproductive System Development and Function	Production of sperm	2.37E-02
Cellular Function and Maintenance	Production of sperm	2.37E-02
Cellular Growth and Proliferation	Production of sperm	2.37E-02
Tissue Morphology	Quantity of macrophages	2.37E-02
Cell Morphology	Size of cells	2.37E-02
Connective Tissue Development and Function	Quantity of connective tissue cells	3.82E-02

*Note*: P values indicate the relevance of the function, with lower values suggesting greater relevance.

**Table 2:** The predicted functions of the DE miRNAs analyzed by ingenuity pathway analysis (IPA).

**Category**	**p-value**	**Number of molecules**
Hematological system development and function	1.3E-03-1.3E-03	3
Tissue morphology	1.3E-03-3.31E-02	19
Connective tissue development and function	1.45E-03-3.31E-02	14
Reproductive system development and function	3.49E-03-4.77E-02	40
Organ morphology	3.92E-03-4.77E-02	38
Cell morphology	1.09E-02-1.8E-02	10
Molecular transport	1.13E-02-4.77E-02	7
Small molecule biochemistry	1.19E-02-4.77E-02	6
Cellular growth and proliferation	2.2E-02-4.01E-02	11
Lipid metabolism	3.24E-02-4.77E-02	6
Cell death and survival	3.31E-02-3.31E-02	2
Cellular function and maintenance	3.31E-02-3.31E-02	2
Drug metabolism	4.77E-02-4.77E-02	3
Endocrine system development and function	4.77E-02-4.77E-02	3

*Note*: P values indicate relevance of the function, with lower values suggesting greater relevance.
