# Supplementary material for: Roles of small RNAs in the effects of nutrition on apoptosis and spermatogenesis in the adult testis
Source: Sci Rep. 2015 May 21;5:10372. doi: 10.1038/srep10372 (PMC4440528; doi:10.1038/srep10372)
Supplement: Supporting Information [file srep10372-s1.pdf]

Roles of small RNAs in the effects of nutrition on apoptosis and spermatogenesis in the adult testis

Yongjuan Guan[1 §], Guanxiang Liang[2 §], Penelope A. R. Hawken[1], Irek A. Malecki[1,4], Greg Cozens[5], Philip E. Vercoe[1], Graeme B. Martin[1,3*], Le Luo Guan[2*]

[1] UWA Institute of Agriculture and School of Animal Biology, University of Western Australia, 35 Stirling Highway, Crawley, WA 6009;

[2] Department of Agricultural, Food and Nutritional Science, University of Alberta, Edmonton, Alberta, T6G 2P5, Canada

[3] Nuffield Department of Obstetrics & Gynaecology, University of Oxford, Oxford OX3 9DU, UK

[4] Department of Animal Sciences, University of Stellenbosch, Matieland 7600, South Africa

[5] School of Anatomy, Physiology and Human Biology, University of Western Australia, 35 Stirling Highway, Crawley, WA 6009

§ These authors contributed equally to the current work

* Corresponding authors

Graeme B. Martin: Tel: +6164886781; Fax: +6164881029; E-mail: graeme.martin@uwa.edu.au.

Leluo Guan: Tel: +17804922480; Fax: +178049244265; E-mail: lguan@ualberta.ca.

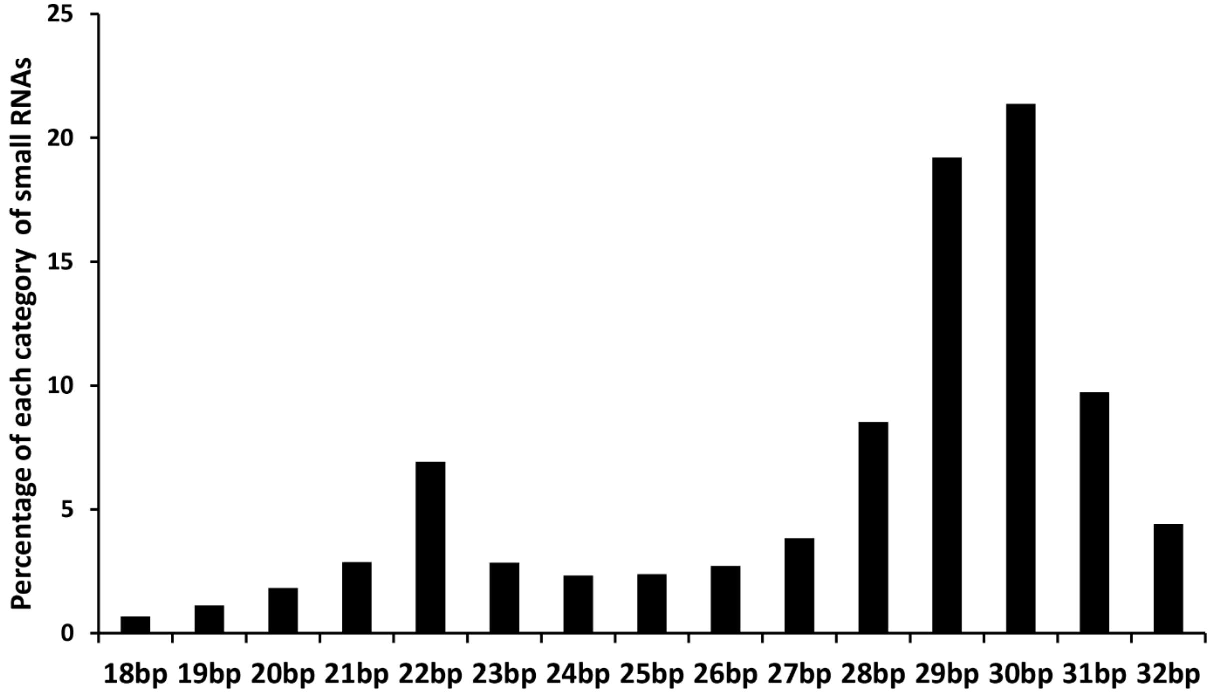

Figure S1. Length distribution of small RNAs in testis from sexually mature male sheep (n = 16). Small RNAs displayed a bimodal length distribution with two peaks at 22 nt and 30 nt.

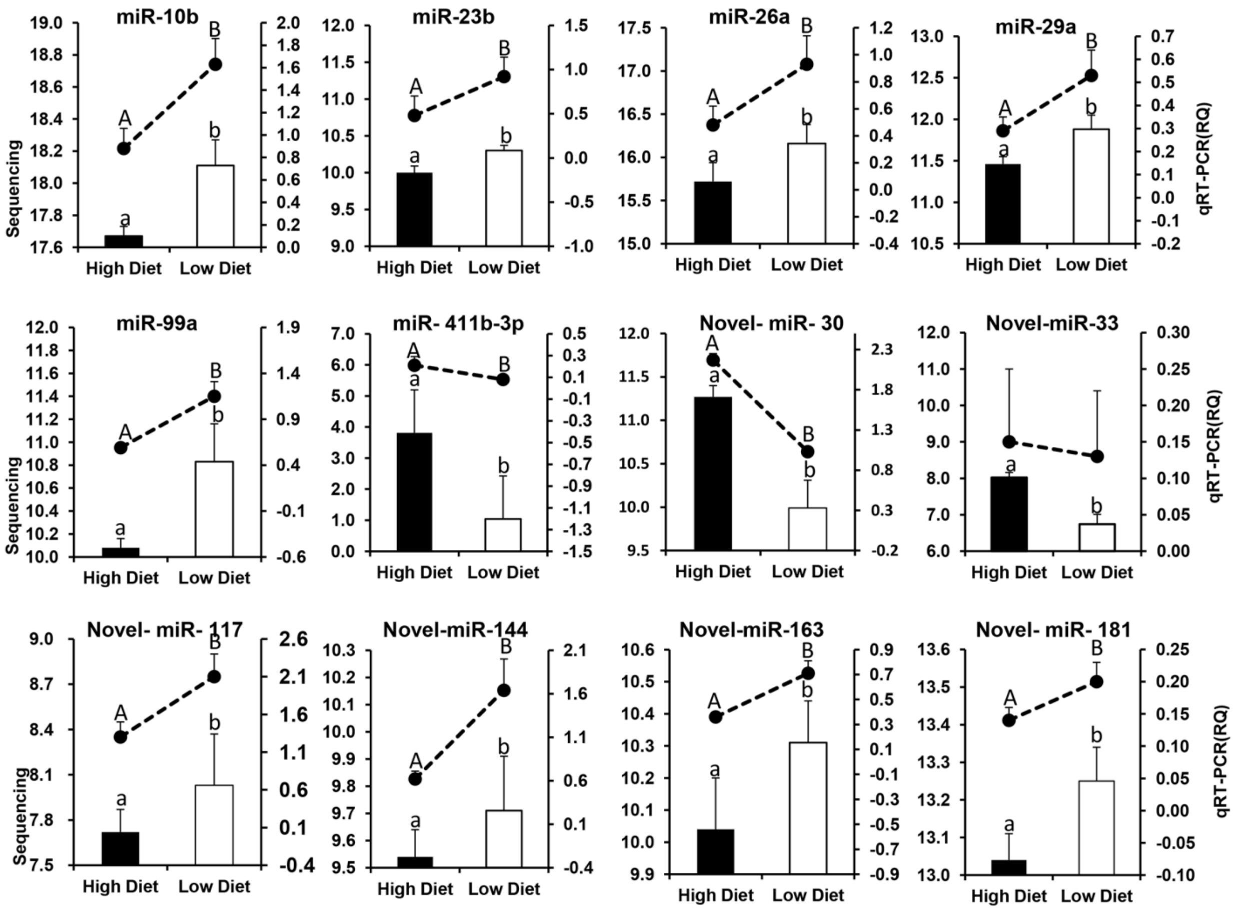

Figure S2. Expression of DE miRNAs in testis from sexually mature sheep fed the High diet or the Low diet, as detected by qRT-PCR and miRNA-seq. Measurement of relative expression by qRT-PCR is shown by line graphs and right Y-axis. Measurement of expression by miRNA-seq is shown by bar graphs and the left Y-axis (values are log2 of normalized number of reads). A, B, C: different letters denote significant difference in the relative expression detected by qRT-PCR; a, b, c: different letters denote significant difference in the expression detected by miRNA-seq. Values are mean ± SE (N = 8 per treatment).

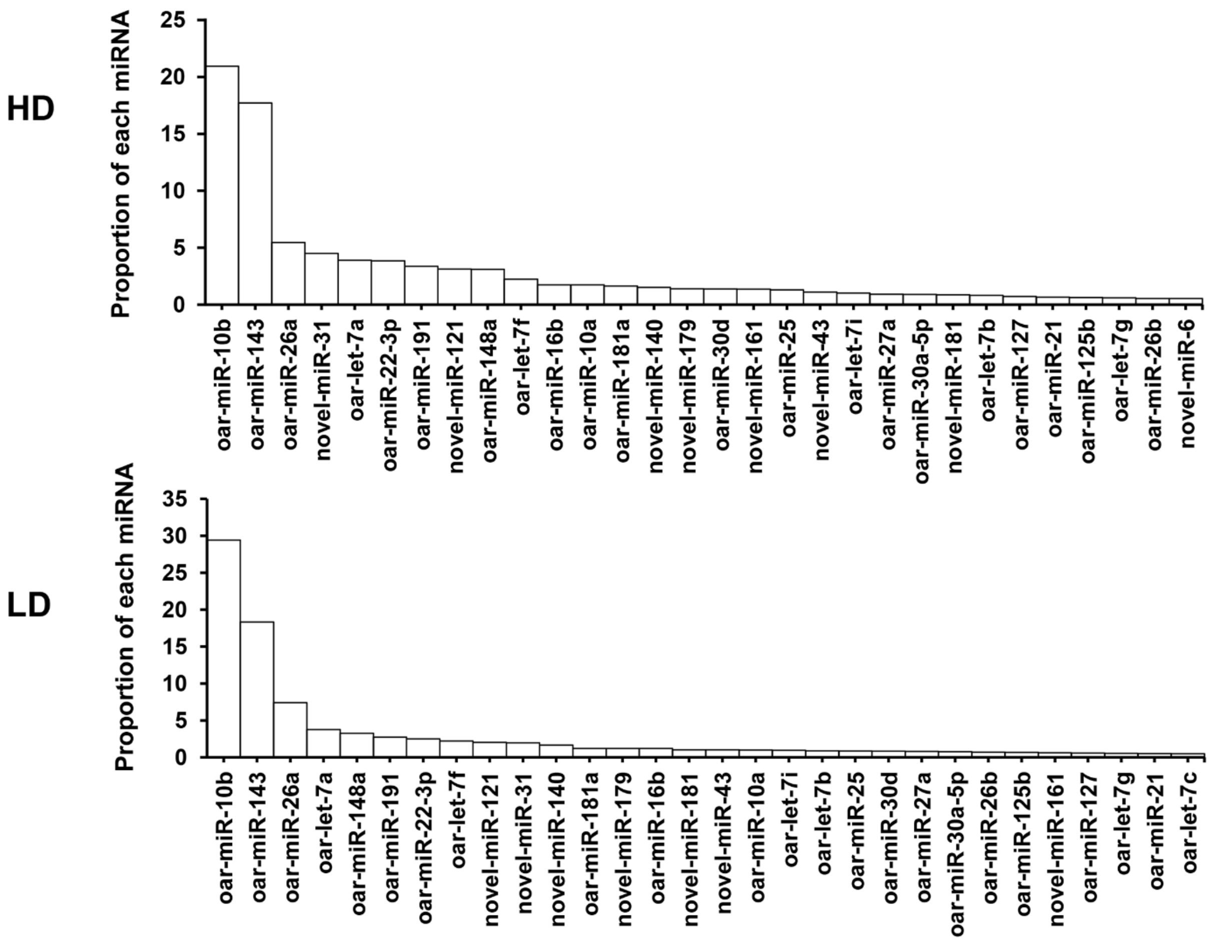

Figure S3. Top 30 highly expressed miRNAs detected in the testis of male sheep fed a high diet (HD) and a low diet (LD). N = 8 for each treatment.

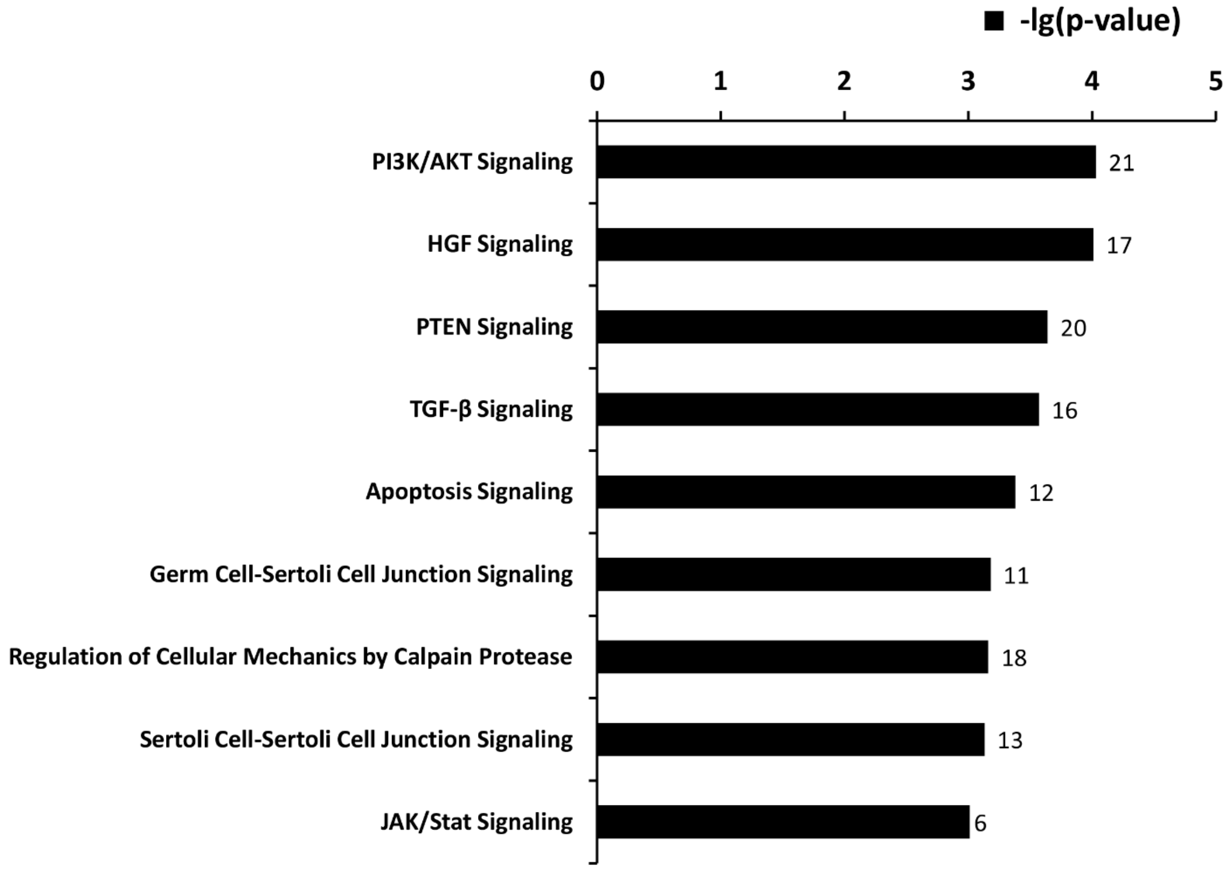

Figure S4. The top 9 signaling pathways of DE miRNAs analyzed by ingenuity pathway analysis (IPA). The X-axis is –lg(p-value) and indicates the relevance of the pathway to the DE miRNAs, with a lower p-value (a higher value of – lg(p-value)) suggesting greater relevance.

Table S1. Details of primers used for RT-QPCR

Gene	Primer Sequence	Product size (bp)	Gene Bank
FASL	F:CCACGTGGCTGGTATCAACT		
	R:GGCTGACAGCAAAACAGGTG	108	NM_001123003.1
TP53	F:GTCATCTAGCGTCCGACCTC		
	R:TGTCTCCTGACTCAGAGGGG	147	NM_001009403.1
CASP3	F:GGCTCTGAGTGTTTGGGGAA		
	R:AGCTCCTGGACAAAGTTCCG	135	XM_004021690.1
GAPDH	F:CTGCTGACGCTCCCATGTTTGT		
	R:TAAGTCCCTCCACGATGCCAAA	150	NM_001190390.1*

Note: * GAPDH primers were obtained from Yu et al. 2010.

Table S2. Details of primers used for amplification of 3'UTR of the predicted targets of novel-miR-144.

Gene	Primer Sequence	Product size (bp)	Gene Bank
FASL	F: CTCGAGAGCACTCTGGGATTCTCTCC		
	R: GTCGACTGCCCTTCCCAATTTCCACAT	694	NM_004013705.1
CASP3	F: CTCGAGCCCAAGGCAAGAAGCTCCA		
	R: GTCGACGGGCTGACATTCAGGGATGG	520	XM_004021690.1
BCL2L1	F: CTCGAGTTCATCCCCACCCTCCAAGA		
	R: GTCGACAGCTGGAAAAAGTGTGGGCT	985	NM_001009226
TP53	F: GTCATCTAGCGTCCGACCTC		
	R: TGTCTCCTGACTCAGAGGGG	895	NM_001009403.1

Table S3. Identification of homologues of novel miRNAs in testis from sexually mature male sheep.

Novel miRNAs	homolog	Conservation
Novel-miR-1	ggo-miR-320a	conserved
Novel-miR-5	cgr-miR-181c-5p	highly conserved
Novel-miR-6	mse-miR-100	highly conserved
Novel-miR-7	cgr-miR-193b-3p	highly conserved
Novel-miR-8	pma-miR-129a-3p	poorly conserved
Novel-miR-11	cgr-miR-1839-5p	poorly conserved
Novel-miR-14	bta-miR-363	highly conserved
Novel-miR-16	aca-miR-214-3p	poorly conserved
Novel-miR-18	pol-miR-140-3p	conserved
Novel-miR-19	bta-miR-1247-5p	poorly conserved
Novel-miR-20	rno-miR-504	conserved
Novel-miR-22	bta-miR-6119-5p	poorly conserved
Novel-miR-23	ssc-miR-219	poorly conserved
Novel-miR-26	cgr-miR-222-3p	highly conserved
Novel-miR-27	cgr-miR-423-3p	poorly conserved
Novel-miR-28	ggo-miR-138	highly conserved
Novel-miR-29	ssc-miR-31	highly conserved
Novel-miR-30	cgr-miR-34b-5p	highly conserved
Novel-miR-31	cgr-miR-34c-5p	highly conserved
Novel-miR-33	ssc-miR-296-3p	conserved
Novel-miR-34	ggo-miR-491	conserved
Novel-miR-38	bta-miR-1388-3p	poorly conserved
Novel-miR-39	cgr-miR-215-5p	highly conserved
Novel-miR-40	ggo-miR-502b	poorly conserved
Novel-miR-41	ggo-miR-628	poorly conserved
Novel-miR-43	cgr-miR-186-5p	conserved
Novel-miR-44	ssc-miR-20b	highly conserved
Novel-miR-45	cgr-miR-93-5p	highly conserved
Novel-miR-46	aca-miR-490-3p	highly conserved
Novel-miR-47	bta-miR-224	conserved
Novel-miR-51	ggo-miR-424	highly conserved
Novel-miR-52	cgr-miR-497-5p	highly conserved
Novel-miR-54	ppy-miR-301b	highly conserved
Novel-miR-55	ccr-miR-130a	poorly conserved
Novel-miR-56	ggo-miR-188	poorly conserved
Novel-miR-57	cgr-miR-532-5p	poorly conserved
Novel-miR-58	rno-miR-1306-5p	poorly conserved
Novel-miR-60	ggo-miR-142	poorly conserved

Novel-miR-63	bta-miR-3431	poorly conserved
Novel-miR-65	eca-miR-326	conserved
Novel-miR-69	bta-miR-2483-5p	poorly conserved
Novel-miR-71	ssc-miR-1468	poorly conserved
Novel-miR-72	ggo-miR-192	highly conserved
Novel-miR-73	ssc-miR-874	conserved
Novel-miR-74	cgr-miR-328	conserved
Novel-miR-75	ccr-miR-129	poorly conserved
Novel-miR-76	bta-miR-6529	poorly conserved
Novel-miR-78	ppy-miR-873	conserved
Novel-miR-80	ggo-miR-331	poorly conserved
Novel-miR-83	eca-miR-345-5p	poorly conserved
Novel-miR-84	bta-miR-503-3p	highly conserved
Novel-miR-87	sha-miR-101	highly conserved
Novel-miR-88	ggo-miR-877	poorly conserved
Novel-miR-89	cgr-miR-505-3p	poorly conserved
Novel-miR-90	pma-miR-145-5p	highly conserved
Novel-miR-91	aca-miR-147	poorly conserved
Novel-miR-93	ccr-miR-20a-5p	highly conserved
Novel-miR-95	cgr-miR-141	poorly conserved
Novel-miR-96	rno-miR-212-3p	highly conserved
Novel-miR-97	ccr-miR-18a	highly conserved
Novel-miR-98	ccr-miR-429	highly conserved
Novel-miR-99	ccr-miR-365	highly conserved
Novel-miR-101	bta-miR-660	poorly conserved
Novel-miR-103	eca-miR-508-5p	poorly conserved
Novel-miR-104	oan-miR-18-3p	poorly conserved
Novel-miR-107	ccr-miR-29a	poorly conserved
Novel-miR-108	cgr-miR-195	highly conserved
Novel-miR-109	cgr-miR-15a-5p	highly conserved
Novel-miR-110	cgr-miR-15b-5p	highly conserved
Novel-miR-111	ccr-miR-196a	highly conserved
Novel-miR-112	dre-miR-196d	highly conserved
Novel-miR-113	ggo-miR-454	highly conserved
Novel-miR-115	ccr-miR-183	highly conserved
Novel-miR-116	ggo-miR-135b	highly conserved
Novel-miR-117	aca-miR-135-5p	highly conserved
Novel-miR-118	cgr-miR-455-5p	highly conserved
Novel-miR-120	cgr-miR-32-5p	highly conserved
Novel-miR-121	ccr-miR-92a	highly conserved
Novel-miR-122	eca-miR-105	highly conserved
Novel-miR-123	bta-miR-105b	poorly conserved
Novel-miR-124	ccr-miR-128	highly conserved

Novel-miR-127	cgr-miR-148b-3p	highly conserved
Novel-miR-128	ccr-miR-338	highly conserved
Novel-miR-129	ssc-miR-339	conserved
Novel-miR-131	cgr-miR-615-3p	conserved
Novel-miR-132	ggo-miR-486	conserved
Novel-miR-134	bta-miR-342	conserved
Novel-miR-135	ppy-miR-330-5p	conserved
Novel-miR-138	pol-miR-9b-5p	highly conserved
Novel-miR-140	ggo-miR-146b	highly conserved
Novel-miR-141	bta-miR-146a	highly conserved
Novel-miR-142	bta-miR-769	poorly conserved
Novel-miR-143	ppy-let-7e	highly conserved
Novel-miR-144	cgr-miR-98	highly conserved
Novel-miR-145	cgr-miR-190a	highly conserved
Novel-miR-146	ggo-miR-190b	highly conserved
Novel-miR-147	eca-miR-507	highly conserved
Novel-miR-149	ppy-miR-767-5p	poorly conserved
Novel-miR-151	bta-miR-6123	poorly conserved
Novel-miR-154	cgr-miR-744-5p	poorly conserved
Novel-miR-156	ccr-miR-7a	highly conserved
Novel-miR-157	ccr-miR-1c	poorly conserved
Novel-miR-158	cgr-miR-184	highly conserved
Novel-miR-161	ppy-miR-449a	highly conserved
Novel-miR-162	ccr-miR-34	highly conserved
Novel-miR-163	cgr-miR-24-3p	highly conserved
Novel-miR-165	bta-miR-30f	highly conserved
Novel-miR-166	sha-miR-30e	highly conserved
Novel-miR-169	ggo-miR-542-3p	conserved
Novel-miR-171	cgr-miR-19a	highly conserved
Novel-miR-173	ccr-miR-499-5p	highly conserved
Novel-miR-174	ppy-miR-155	highly conserved
Novel-miR-175	mml-miR-1296	poorly conserved
Novel-miR-176	cgr-miR-340-5p	conserved
Novel-miR-177	ggo-miR-361-5p	conserved
Novel-miR-178	rno-miR-3585-5p	poorly conserved
Novel-miR-179	ggo-miR-27b	highly conserved
Novel-miR-180	ggo-miR-197	conserved
Novel-miR-181	cgr-miR-204	highly conserved
Novel-miR-183	pma-miR-153-3p	highly conserved
Novel-miR-186	ppy-miR-592	conserved
Novel-miR-188	mmu-miR-670-3p	poorly conserved
Novel-miR-191	pol-miR-133-3p	poorly conserved
Novel-miR-193	cgr-miR-450a	poorly conserved
Novel-miR-194	mmu-miR-335-3p	poorly conserved

Table S4. Identification of clustered miRNAs in testis from sexually mature male sheep. Note: Fold change (FC) = CPM of low diet group/CPM of high diet group. CPM (counts per million) = (piRNAs reads number/total reads number per library) × 1,000,000. The significant DE piRNAs were determined by false discovery rate (FDR) < 0.05.

Cluster	miRNA precusors	Chromosome	Start	End	Chain	Fold change	FDR
1	oar-let-7c	1	138828281	138828348	-	1.584886515	0.12
1	oar-mir-99a	1	138829044	138829104	-	1.980160098	0.00
2	oar-let-7d	2	27312334	27312420	-	1.362595936	0.12
2	oar-let-7f	2	27314541	27314619	-	1.232809484	0.82
3	oar-mir-23a	2	31139258	31139316	-	1.526131083	0.17
3	oar-mir-23b	2	31139258	31139316	-	1.715777266	0.01
4	oar-mir-29a	4	94625756	94625815	-	1.894866859	0.00
4	oar-mir-29b	4	94626148	94626212	-	1.848852521	0.15
5	Novel-mir-97	10	66183231	66183294	+	0.863758469	0.72
5	Novel-mir-171	10	66183379	66183435	+	1.973624423	0.25
5	Novel-mir-93	10	66183541	66183600	+	1.067442176	0.82
5	Novel-mir-121	10	66183794	66183853	+	0.811971043	0.29
6	oar-mir-200a	12	49397310	49397370	-	3.11106299	0.06
6	oar-mir-200b	12	49397879	49397938	-	2.385865368	0.07
7	Novel-mir-151	13	56861602	56861671	+	0.620328913	0.12
7	Novel-mir-33	13	56861865	56861922	+	0.525667371	0.02
8	oar-mir-431	18	64480275	64480365	+	NA	NA
8	oar-mir-433-3p	18	64481132	64481245	+	NA	NA
8	oar-mir-127	18	64482256	64482312	+	1.035213089	1.00
8	oar-mir-432	18	64483745	64483814	+	0.639252679	0.11
8	oar-mir-136	18	64483932	64483988	+	0.977629258	0.32
9	oar-mir-299-5p	18	64620136	64620188	+	0.582366793	0.04
9	oar-mir-299-3p	18	64620136	64620188	+	NA	NA
9	oar-mir-380-3p	18	64621337	64621394	+	0.566096672	0.06
9	oar-mir-411b-3p	18	64621498	64621554	+	0.248428524	0.02
9	oar-mir-1197-5p	18	64621814	64621942	+	NA	NA
9	oar-mir-1197-3p	18	64621814	64621942	+	0.537416643	0.05
9	oar-mir-323a-3p	18	64622016	64622072	+	0.456090204	0.00
9	oar-mir-758-3p	18	64622312	64622370	+	0.534382926	0.06
9	oar-mir-329b-3p	18	64623030	64623089	+	0.847076851	0.59
9	oar-mir-494-3p	18	64626212	64626270	+	0.476289717	0.02
9	oar-mir-543-3p	18	64628562	64628619	+	0.660922349	0.60
9	oar-mir-495-3p	18	64629999	64630057	+	0.968098552	0.81
9	oar-mir-3958-5p	18	64631942	64631998	+	0.429111578	0.32

9	oar-mir-3958-3p	18	64631942	64631998	+	0.402147109	0.01
9	oar-mir-376b-3p	18	64634621	64634681	+	0.90351894	0.79
9	oar-mir-376c-3p	18	64634992	64635050	+	0.919212574	0.89
9	oar-mir-376d	18	64635363	64635421	+	0.82520239	0.94
9	oar-mir-376e-3p	18	64635739	64635798	+	0.678302164	0.07
9	oar-mir-376a-3p	18	64636085	64636187	+	0.471194039	0.05
9	oar-mir-1185-5p	18	64638058	64638118	+	0.602903914	0.03
9	oar-mir-1185-3p	18	64638058	64638118	+	0.50697974	0.02
9	oar-mir-381-3p	18	64639738	64639801	+	0.517586244	0.06
9	oar-mir-381-3p	18	64639738	64639801	+	0.517586244	0.06
9	oar-mir-487b-3p	18	64640234	64640291	+	0.437432822	0.24
9	oar-mir-539-3p	18	64641003	64641061	+	0.678302164	0.36
9	oar-mir-544-5p	18	64642597	64642697	+	0.435275282	0.04
9	oar-mir-655-3p	18	64643523	64643583	+	1.110827145	0.82
9	oar-mir-3959-5p	18	64645543	64645597	+	0.616192452	0.26
9	oar-mir-3959-3p	18	64645543	64645597	+	0.48856939	0.90
9	oar-mir-487a-3p	18	64645957	64646015	+	0.494240374	0.06
9	oar-mir-382-5p	18	64648908	64648965	+	0.856893187	0.30
9	oar-mir-382-3p	18	64648908	64648965	+	0.515520369	0.25
9	oar-mir-134-5p	18	64649271	64649334	+	0.624165274	0.04
9	oar-mir-485-5p	18	64649969	64650028	+	0.56949063	0.82
9	oar-mir-485-3p	18	64649969	64650028	+	0.58179404	0.27
10	Novel-mir-7	24	13219122	13219181	+	1.380577355	0.72
10	Novel-mir-99	24	13223800	13223862	+	1.369002591	0.30
11	Novel-mir-101	X	52158377	52158433	-	1.2288107	0.45
11	Novel-mir-40	X	52160489	52160551	-	1.164573195	0.58
11	Novel-mir-56	X	52175957	52176017	-	1.210895151	0.91
11	Novel-mir-57	X	52176280	52176339	-	1.594851755	0.10
12	Novel-mir-123	X	78722115	78722173	+	0.590118399	0.69
12	Novel-mir-149	X	78723038	78723094	+	0.587297102	0.07
12	Novel-mir-122	X	78724306	78724365	+	0.74238861	0.29
13	Novel-mir-47	X	79046399	79046467	+	1.251729498	0.12
13	Novel-mir-63	X	79048337	79048399	+	1.606445935	0.11
14	Novel-mir-105	X	84537948	84538004	+	0.515520369	0.20
14	Novel-mir-17	X	84538978	84539035	+	0.426811782	0.04
15	Novel-mir-51	X	95343715	95343773	+	1.351991113	0.33
15	Novel-mir-84	X	95344046	95344109	+	1.048140434	1.00
15	Novel-mir-169	X	95348844	95348903	+	1.157054382	0.58
15	Novel-mir-193	X	95350011	95350066	+	1.51624386	0.13
16	Novel-mir-104	X	95607969	95608031	+	1.572303012	0.47
16	Novel-mir-44	X	95608208	95608268	+	0.930382533	0.82
16	Novel-mir-121	X	95608483	95608544	+	0.811971043	0.29
16	Novel-mir-14	X	95608637	95608702	+	0.926128863	0.94

Table S5. Differentially expressed piRNAs in testis from sheep fed a low or high diet (N = 8 for each treatment). *Note*: Fold change (FC) = CPM of low diet group/CPM of high diet group. CPM (Counts per million) = (piRNAs reads number/total reads number per library) × 1,000,000. The significant DE piRNAs were determined by false discovery rate (FDR) < 0.05.

	logFC	logCPM	PValue	FDR
oar-piR-789	3.64	6.85	6.67E-17	3.33E-13
oar-piR-3085	-3.79	5.96	1.76E-14	4.39E-11
oar-piR-11578	3.50	4.07	6.88E-13	1.15E-09
oar-piR-6442	4.29	3.59	1.88E-11	2.35E-08
oar-piR-606	2.19	6.72	2.32E-10	2.32E-07
oar-piR-12439	-1.94	8.65	1.30E-09	1.08E-06
oar-piR-13207	2.10	5.03	1.32E-07	9.43E-05
oar-piR-2287	-1.98	8.89	2.06E-07	1.28E-04
oar-piR-1866	-2.06	4.89	3.68E-07	1.93E-04
oar-piR-6716	-2.57	4.41	3.85E-07	1.93E-04
oar-piR-3404	-2.91	5.14	5.10E-07	2.30E-04
oar-piR-11406	1.73	5.75	5.84E-07	2.30E-04
oar-piR-1617	-2.38	6.06	5.99E-07	2.30E-04
oar-piR-2194	-1.56	5.21	7.53E-07	2.69E-04
oar-piR-3337	-3.28	5.07	1.35E-06	4.50E-04
oar-piR-10216	2.45	3.93	1.95E-06	5.75E-04
oar-piR-10217	2.45	3.93	1.95E-06	5.75E-04
oar-piR-10729	2.13	4.19	3.02E-06	7.54E-04
oar-piR-10730	2.13	4.19	3.02E-06	7.54E-04
oar-piR-10731	2.13	4.19	3.02E-06	7.54E-04
oar-piR-2327	-2.20	8.53	3.36E-06	8.00E-04
oar-piR-300	-1.47	6.89	3.63E-06	8.24E-04
oar-piR-12568	0.56	6.63	3.90E-06	8.48E-04
oar-piR-2936	1.54	5.18	4.56E-06	9.50E-04
oar-piR-664	1.61	7.59	2.14E-05	4.28E-03
oar-piR-9120	-2.26	4.63	3.90E-05	7.49E-03
oar-piR-9006	-1.05	5.34	4.68E-05	8.53E-03
oar-piR-6573	1.54	6.39	4.78E-05	8.53E-03
oar-piR-223	-0.93	11.98	5.02E-05	8.65E-03
oar-piR-644	-1.98	4.11	6.04E-05	1.01E-02
oar-piR-2747	1.52	5.23	7.48E-05	1.21E-02
oar-piR-1189	-0.89	10.55	1.33E-04	2.07E-02
oar-piR-2886	1.13	9.77	1.42E-04	2.14E-02
oar-piR-7322	1.38	5.99	1.59E-04	2.34E-02
oar-piR-1248	-1.06	5.35	3.32E-04	4.74E-02

Table S6. Gene-derived DE piRNAs in testis from sexually mature male sheep fed a high or low diet (N = 8 for each treatment).

piRNA	Genomic Location	Gene full name	Gene abbreviation name
oar-piR-12568	3'UTR	feline leukemia virus subgroup C cellular receptor family, member 2	FLVCR2
oar-piR-6442	3'UTR	keratin associated protein 10-2	KRTAP10-2
oar-piR-9006	5'UTR	ATPase, Ca++ Transporting, Plasma Membrane 4	ATP2B4
oar-piR-10216	Intron	Mortality Factor 4 Like 1	MORF4L1
oar-piR-10217	Intron	Mortality Factor 4 Like 1	MORF4L1
oar-piR-10729	Intron	Mortality Factor 4 Like 1	MORF4L1
oar-piR-10730	Intron	Mortality Factor 4 Like 1	MORF4L1
oar-piR-10731	Intron	Mortality Factor 4 Like 1	MORF4L1
oar-piR-11406	Intron	sorting nexin 5	SNX5
oar-piR-1248	Intron	Storkhead-Box Protein 1	STOX1
oar-piR-2194	Intron	ENSOARG00000013508	ENSOARG00000013508
oar-piR-2936	Intron	fumarylacetoacetate hydrolase	FAH
oar-piR-7322	Intron	C-Type Lectin Domain Family 16, Member	CLEC16A
oar-piR-9006	Intron	DDB1 And CUL4 Associated Factor 6	DCAF6
